# Mild intracellular acidification by dexamethasone attenuates mitochondrial dysfunction in a human inflammatory proximal tubule epithelial cell model

**DOI:** 10.1038/s41598-017-10483-y

**Published:** 2017-09-06

**Authors:** T. J. J. Schirris, J. Jansen, M. Mihajlovic, L. P. van den Heuvel, R. Masereeuw, F. G. M. Russel

**Affiliations:** 1 Department of Pharmacology and Toxicology, Radboud University Medical Center, Radboud Institute for Molecular Life Sciences, 6500 HB Nijmegen, The Netherlands; 2 Center for Systems Biology and Bioenergetics, Radboud Center for Mitochondrial Medicine, Radboud University Medical Center, 6500 HB Nijmegen, The Netherlands; 3 Department of Physiology, Radboud University Medical Center, Radboud Institute for Molecular Life Sciences, 6500HB Nijmegen, The Netherlands; 4 Department of Pediatrics, Radboud University Medical Center, 6500 HB Nijmegen, The Netherlands; 5 Division of Pharmacology, Utrecht Institute for Pharmaceutical Sciences, 3584 CG Utrecht, The Netherlands; 6 Department of Pediatric Nephrology & Growth and Regeneration, Catholic University Leuven, 3000 Leuven, Belgium

## Abstract

Septic acute kidney injury (AKI) associates with poor survival rates and often requires renal replacement therapy. Glucocorticoids may pose renal protective effects in sepsis via stimulation of mitochondrial function. Therefore, we studied the mitochondrial effects of dexamethasone in an experimental inflammatory proximal tubule epithelial cell model. Treatment of human proximal tubule epithelial cells with lipopolysaccharide (LPS) closely resembles pathophysiological processes during endotoxaemia, and led to increased cytokine excretion rates and cellular reactive oxygen species levels, combined with a reduced mitochondrial membrane potential and respiratory capacity. These effects were attenuated by dexamethasone. Dexamethasone specifically increased the expression and activity of mitochondrial complex V (CV), which could not be explained by an increase in mitochondrial mass. Finally, we demonstrated that dexamethasone acidified the intracellular milieu and consequently reversed LPS-induced alkalisation, leading to restoration of the mitochondrial function. This acidification also provides an explanation for the increase in CV expression, which is expected to compensate for the inhibitory effect of the acidified environment on this complex. Besides the mechanistic insights into the beneficial effects of dexamethasone during renal cellular inflammation, our work also supports a key role for mitochondria in this process and, hence, provides novel therapeutic avenues for the treatment of AKI.

## Introduction

The prevalence of acute kidney injury (AKI) in critically ill patients has rapidly increased over the past two decades up to 30 to 40% of patients admitted to the intensive care unit^1, 2^. Overall, AKI is associated with 20% of all hospitalised adults worldwide, which results in a high burden on healthcare^3^. Though depending on AKI severity and cause (*i.e*. prerenal, intrinsic or postrenal), the mortality rate is over 50%, and even less severe manifestations are associated with short- and long-term adverse effects, including chronic kidney disease^4^. The pathogenesis of AKI is highly complex and often multi-causal, but the septic form provoked by endotoxins originating from gram-negative bacteria is the most common cause of disease onset^5^.

Currently, no pharmacological therapeutic interventions are approved to prevent or treat AKI. Therefore, treatment is limited to mitigating secondary hemodynamic and toxic renal insults and provision of supportive measures such as diuretics and renal replacement therapy, predominantly hemodialysis^1^. A variety of renal protective mechanisms have been explored *in vitro* for their therapeutic potency in AKI (*e.g*. antioxidant, anti-inflammatory, or anti-apoptotic effects, or the activation of autophagy)^6^. Still, most of these strategies have not yet reached clinical studies, and none are applied in a clinical setting^7^. Promising clinical effects have recently been observed with alkaline phosphatase (AP) in two phase-II trials that demonstrated improved kidney function in critically ill patients with sepsis-associated AKI^8^. Mechanistic studies further showed that AP dephosphorylates extracellular ATP and ADP being released after lipopolysaccharide (LPS) exposure, which might be key in the renal protective effect^8^. However, further clinical validation should demonstrate the true efficacy and efficiency of AP prior to clinical implementation. Another potential replacement therapy includes a bioartificial kidney containing renal proximal tubule epithelial cells to correct uraemia. Studies in animal models and patients with AKI demonstrated immunomodulatory effects, though controlled randomised multi-centre human studies have not yet shown conclusive beneficial evidence^9–11^.

Recently, treatment of cardiac surgery patients with the glucocorticoid dexamethasone has been proposed as a new strategy against AKI^12, 13^. Dexamethasone-induced attenuation of septic AKI was also demonstrated in several *in vitro* and *in vivo* models, including diminished cytokine levels, improved glomerular filtration rate, suppressed pro-apoptotic proteins and reduced mitochondrial injury, inhibited inducible nitric oxide (NO) synthase (iNOS) activity, and improved fluid balance^14–18^. In addition, stimulation of multi drug resistance protein-2 (MRP2) was observed, resulting in enhanced urinary excretion of endo- and xenobiotics^19^.

Still, the exact molecular mechanism underlying these beneficial effects remains unknown. Increasing evidence points towards the role of mitochondrial dysfunction in the pathophysiology of AKI^3, 16, 20^, which is in agreement with mitochondrial dependence of the proximal as well as the distal nephron segments, as illustrated by the high density of these organelles^21^. Mitochondrial dysfunction was characterised by a decreased cytochrome *c* oxidase (COX; mitochondrial complex IV) expression, most likely under the regulation of increased expression of Bcl-2 pro-apoptotic proteins^16^, and elevated reactive oxygen species (ROS). COX and Bcl-2 protein expression were partially restored upon dexamethasone treatment, suggesting a potential mitochondrial mechanism^16^. In addition, peroxisome proliferator-activated receptor gamma (PPARγ) coactivator - 1α (PGC-1α), a known key regulator of mitochondrial biogenesis and predominantly expressed in proximal tubules, was suppressed^20, 22^. Upon recovery, PGC-1α was restored, emphasising the pivotal role of mitochondria to treat AKI. However, further elucidation of the exact mechanism by which dexamethasone influences mitochondrial homeostasis pathways during AKI is required.

To this end, we used human conditionally immortalised proximal tubule epithelial cells (ciPTEC, ref. 23). We previously characterised this model and demonstrated a broad range of PTEC-specific transport and metabolic functions^23^. Cells were challenged with LPS to mimic septic AKI and were co-incubated with dexamethasone, which improved mitochondrial respiration, mitochondrial respiratory complex activity, membrane potential, and attenuated ROS production. These effects of dexamethasone were not mediated via PGC-1α-induced mitochondrial biogenesis, but likely associated with a restoration of cellular pH.

## Results

### Dexamethasone attenuates LPS-induced endotoxemia associated mitochondrial dysfunction

**Figure 1:**
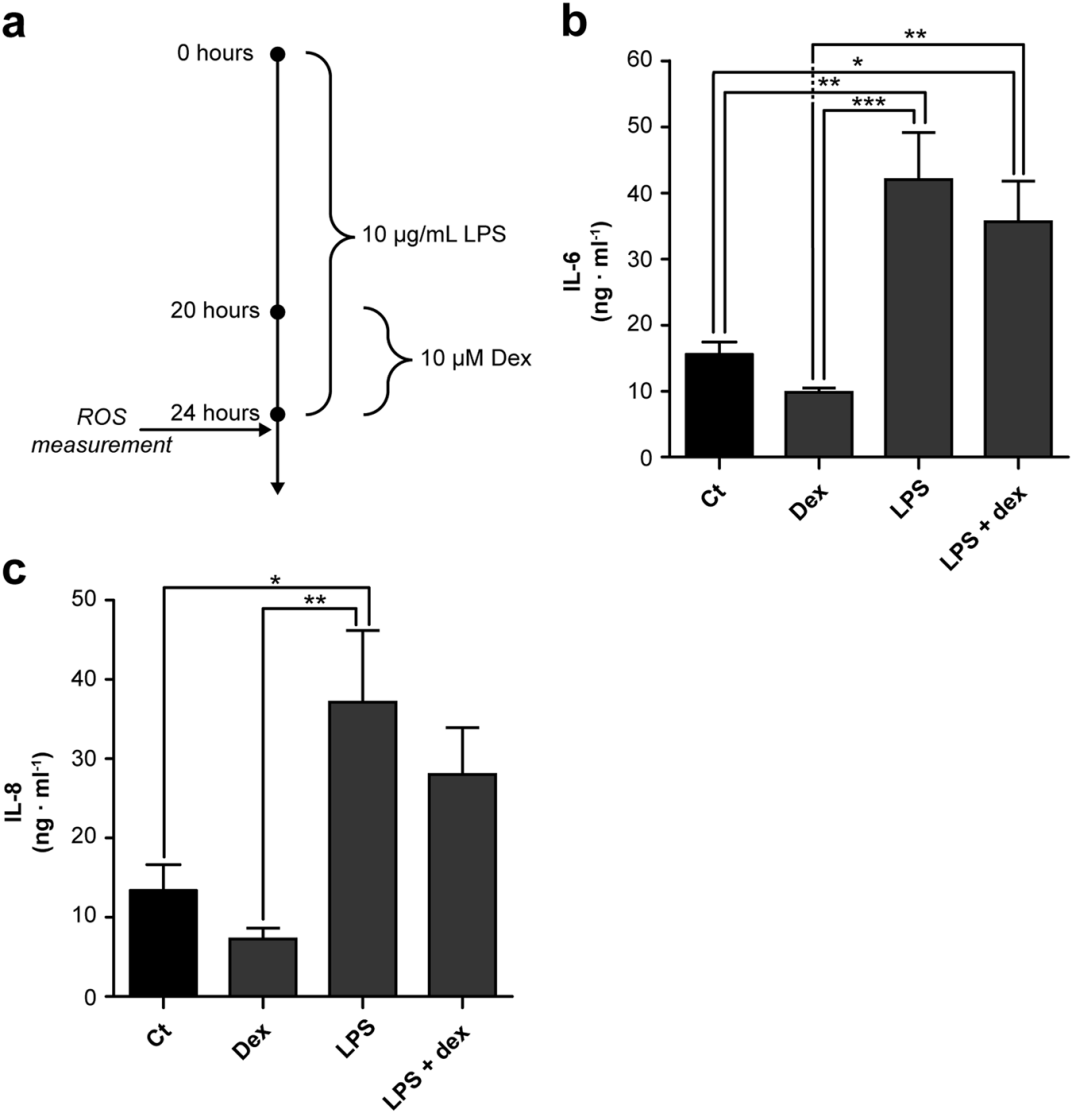
Dexamethasone co-treatment reduced IL-6 and IL-8 cytokine production in LPS-induced endotoxsemia. (**a)** To induce endotoxaemia, ciPTEC were exposed to 10 μg/mL lipopolysacharide (LPS) for 20 hours. Subsequently, the cells were co-treated with dexamethasone (10 μM, Dex) for 4 hours, and **(b)** interleukin **(**IL)-6 and **(c)** IL-8 cytokine excretion into the culture medium was measured by ELISA. Statistical analysis: one-way ANOVA with Tukey’s post-hoc analysis compared to vehicle control (Ct): *p < 0.05, **p < 0.01, ***p < 0.001. Mean ± SEM, n = 5 independent experiments.

Production of interleukin (IL)-6 and IL-8 in renal cells was clearly stimulated upon twenty-four-hour LPS treatment (42 ± 7 ng·ml^−1^, p = 0.0016 vs. 37 ± 9 ng·ml^−1^, p < 0.025, respectively) (Fig. 1a–c), thereby mimicking the inflammatory response observed *in vivo*. This is in agreement with our recent studies on sepsis-induced endotoxemia using ciPTEC^8^. Upon co-treatment with dexamethasone, the IL-6 production was not altered (Fig. 1b), however, IL-8 production was no longer significantly increased compared to vehicle-treated cells (Fig. 1c).

**Figure 2:**
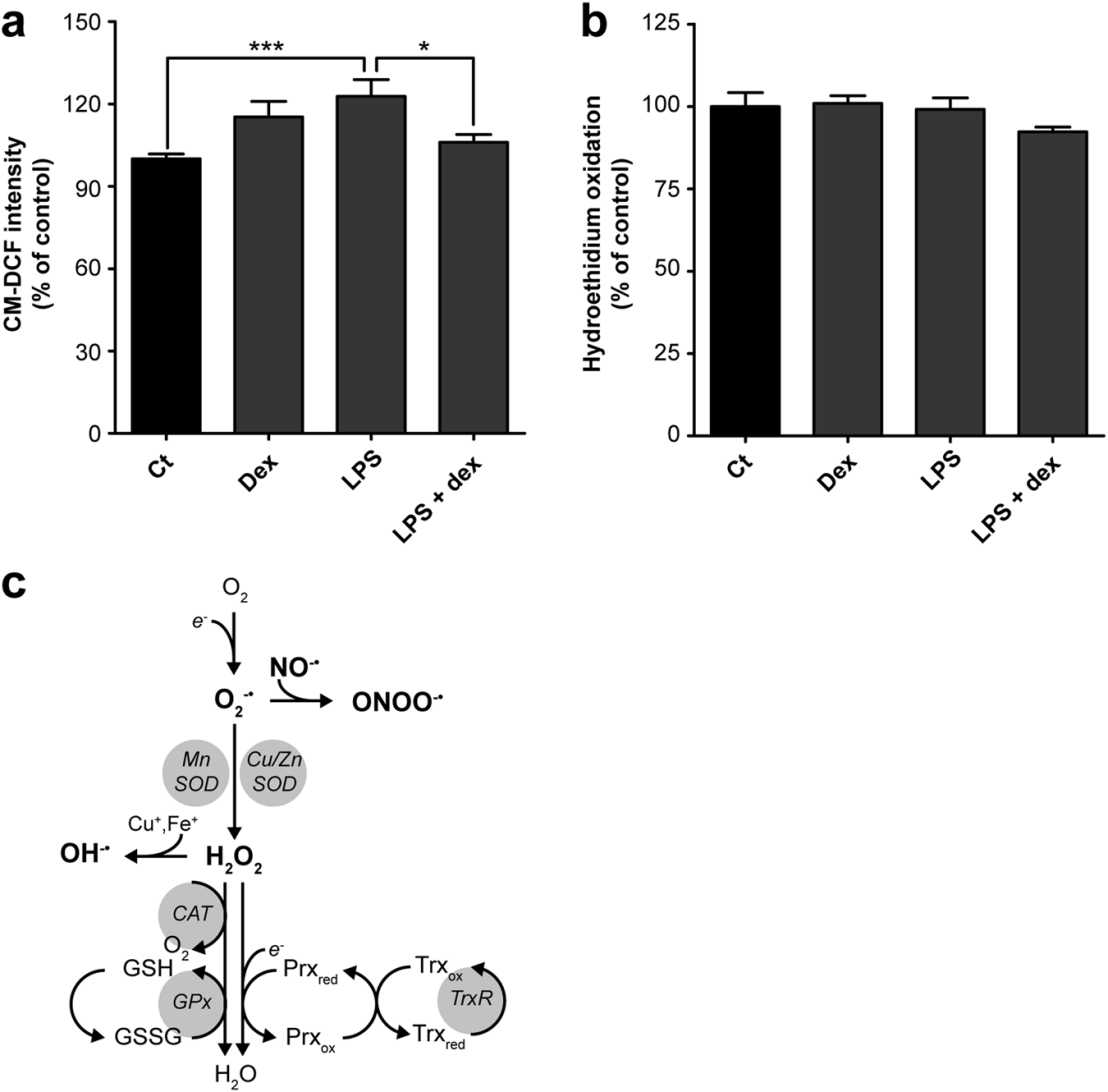
LPS-induced endotoxaemia is characterised by ROS generation and is attenuated by dexamethasone treatment. After dexamethasone and/or LPS exposure (Fig. 1a) the generation of reactive oxygen species (ROS) is examined using **(a)** 5-(and-6)-chloromethyl-2′,7′-dichlorodihydrofluorescein diacetate, acetyl ester (CM-DCF) or **(b)** hydroethidium for the detection of general cellular ROS species or superoxide anions (O_2_^−^^•^), respectively. Values were normalised to control (Ct): CM-DCF (766 ± 64 arbitrary intensity units), hydroethidium (343 ± 64 arbitrary intensity units). **(c)** Cellular fate of superoxide anions into reactive nitrogen species (NO^−^^•^, ONOO^−^^•^), hydroxyl radicals (OH^−^^•^) and hydrogen peroxide (H_2_O_2_), which is catalysed by different enzymes (grey spheres), including manganese or copper/zinc superoxide dismutase (MnSOD, Cu/ZnSOD), catalase (CAT), glutathione peroxidase (GPx), and the peroxidoxin/thioredoxin (Prx/Trx) system catalysed by thioredoxin reductase (TrxR). Statistic analysis: one-way ANOVA with Tukey’s post-hoc analysis: *p < 0.05, ***p < 0.001. Mean ± SEM, n = 3 independent experiments.

**Figure 3:**
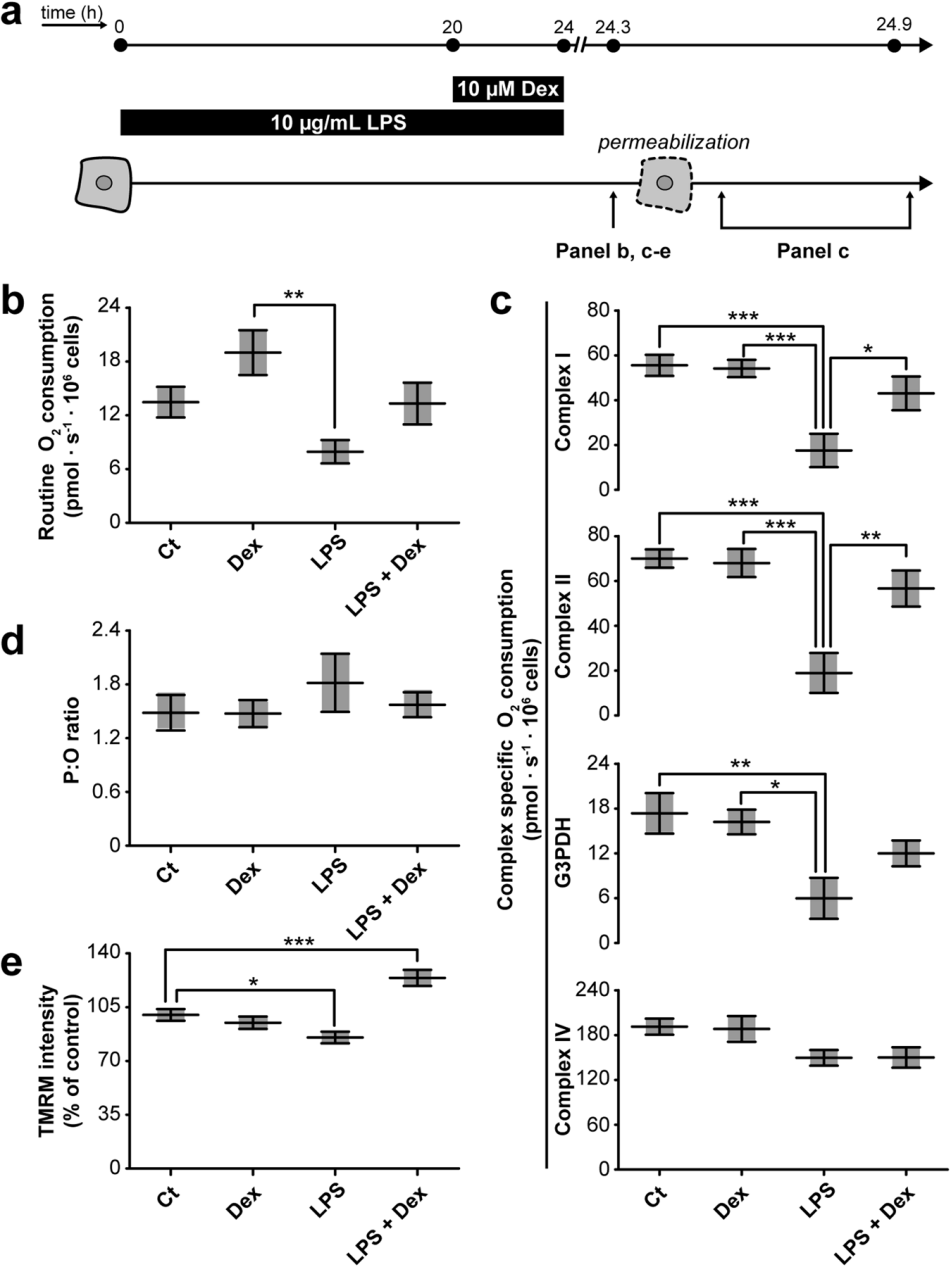
LPS-induced endotoxaemia leads to severe mitochondrial dysfunction that is restored by dexamethasone treatment. (**a)** Cells were treated with LPS and/or dexamethasone as described in detail in Fig. 1a. (**b**) Next, cells were harvested and transferred to the chambers of the respirometer to determine routine oxygen consumption. **(c)** After permeabilisation of the plasma membrane, OXPHOS complex I (CI)-, CII-, glycerol-3-phosphate dehydrogenase (G3PDH)-, and CIV-driven oxygen consumption rates were measured. **(d)** Before measurement of these complex-specific respiratory rates the mitochondrial coupling (*e.g*. coupling between the respiratory chain, ATP production and mitochondrial membrane potential) was determined using the phosphorus:oxygen (P:O) ratio. This ratio was calculated dividing the amount of ADP used by the amount of oxygen consumed to use all the ADP (220 nM). **(e)** Alternatively, mitochondrial coupling was determined by directly measuring the mitochondrial membrane potential using the cationic dye tetramethylrhodamine methyl ester (TMRM). Values were normalised to control (Ct): 158 ± 22 arbitrary intensity units. Statistic analysis: one-way ANOVA with Tukey’s post-hoc analysis: *p < 0.05, **p < 0.01, ***p < 0.001. Mean ± SEM, n = 3 independent experiments.

Next, we further investigated the cellular responses to LPS by evaluating ROS generation. Exposure of ciPTEC to LPS for 24 hours led to increased ROS levels (123 ± 6%, p < 0.001), using a general ROS indicator (Fig. 2a). However, increased ROS production was not reflected in elevated mitochondrial superoxide anions levels (Fig. 2b). This can be explained by the rapid degradation of superoxide anions into hydrogen peroxide by manganese superoxide dismutase MnSOD or Cu/ZnSOD, and subsequent detoxification, either by the peroxidoxin/thioredoxin system, catalase, or via the reduction of glutathione (GSH) by glutathione peroxidase (Fig. 2c)^24, 25^. After 24-hour incubation, dexamethasone dose-dependently decreased ROS levels **(**Supplementary Fig. 1a **)**, but high concentrations altered the cellular morphology indicative for cytotoxic effects **(**Supplementary Fig. 1b–d **)**. To avoid such toxic effects we used a low (10 μM) concentration, and to even further minimise the chance on adverse effects we combined it with a brief four-hour co-treatment of the cells. This was already sufficient to reverse the LPS-induced ROS generation (Fig. 2a), and respiratory inhibition by increasing oxidative phosphorylation (OXPHOS) CI- (25 ± 9%, p < 0.031) and mitochondrial complex II (CII)-driven respiration (38 ± 11%, p < 0.0046) compared to LPS-treated cells (Fig. 3a–c). Of note, CIV-driven respiration was not reduced after LPS treatment, which could be explained by the auto-oxidative potential of the substrates used (*i.e*. ascorbate and *N,N,N*′*,N*′-tetramethyl-*p*-phenylenediamine (TMPD)) leading to a decreased sensitivity (Fig. 3c). Importantly, respiratory inhibition by LPS was only apparent upon maximal stimulation of the respiratory chain in permeabilised cells, as observed for the respiration driven by CI (32 ± 13%, p = 0.00041), CII (27 ± 13%, p = 0.000061), and glycerol-3-phosphate dehydrogenase (G3PDH; 34 ± 16%, p = 0.0087) respiration, but not under basal conditions (Fig. 3b,c). Such a specific pattern points to a lower mitochondrial respiratory reserve capacity because of a reduced mitochondrial membrane potential or a decreased substrate availability. The ATP produced per oxygen atom reduced by the OXPHOS system (*i.e*. phosphate: oxygen (P:O) ratio), provides an indication for the mitochondrial coupling of both processes. The tendency of LPS to increase the P:O is in line with a decreased coupling (Fig. 3d), which was confirmed by the mitochondrial membrane potential (Fig. 3e). Dexamethasone co-exposure counterbalanced the LPS-induced depolarisation, corroborating the other beneficial effects on mitochondrial function, and even showed hyperpolarisation (Fig. 3e; 124 ± 5%, p = 0.00012).

### Dexamethasone increases OXPHOS complex V activity and its expression

**Figure 4:**
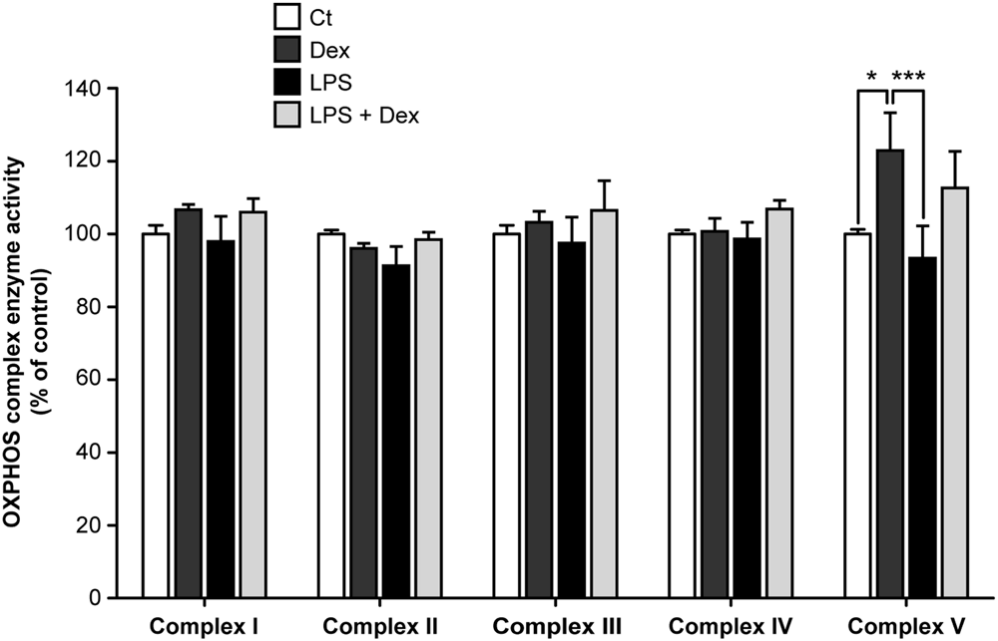
Dexamethasone leads to an increased enzyme activity of OXPHOS complex V without affecting the activity of complex I-IV. After LPS and dexamethasone (Fig. 1a) exposure cells were harvested and complex I-V (CI-CV) enzyme activity was measured spectrophotometrically. Values were corrected for cellular protein and normalised to control (Ct): CI (160 ± 9 U·mg protein^−1^), CII (220 ± 7 U·mg protein^−1^), CIII (202 ± 6 U·mg protein^−1^), CIV (183 ± 10 U·mg protein^−1^), CV (282 ± 87 U·mg protein^−1^). Statistic analysis: two-way ANOVA with Bonferroni’s post-hoc analysis: *p < 0.05, ***p < 0.001. Mean ± SEM, n = 3 independent experiments.

**Figure 5:**
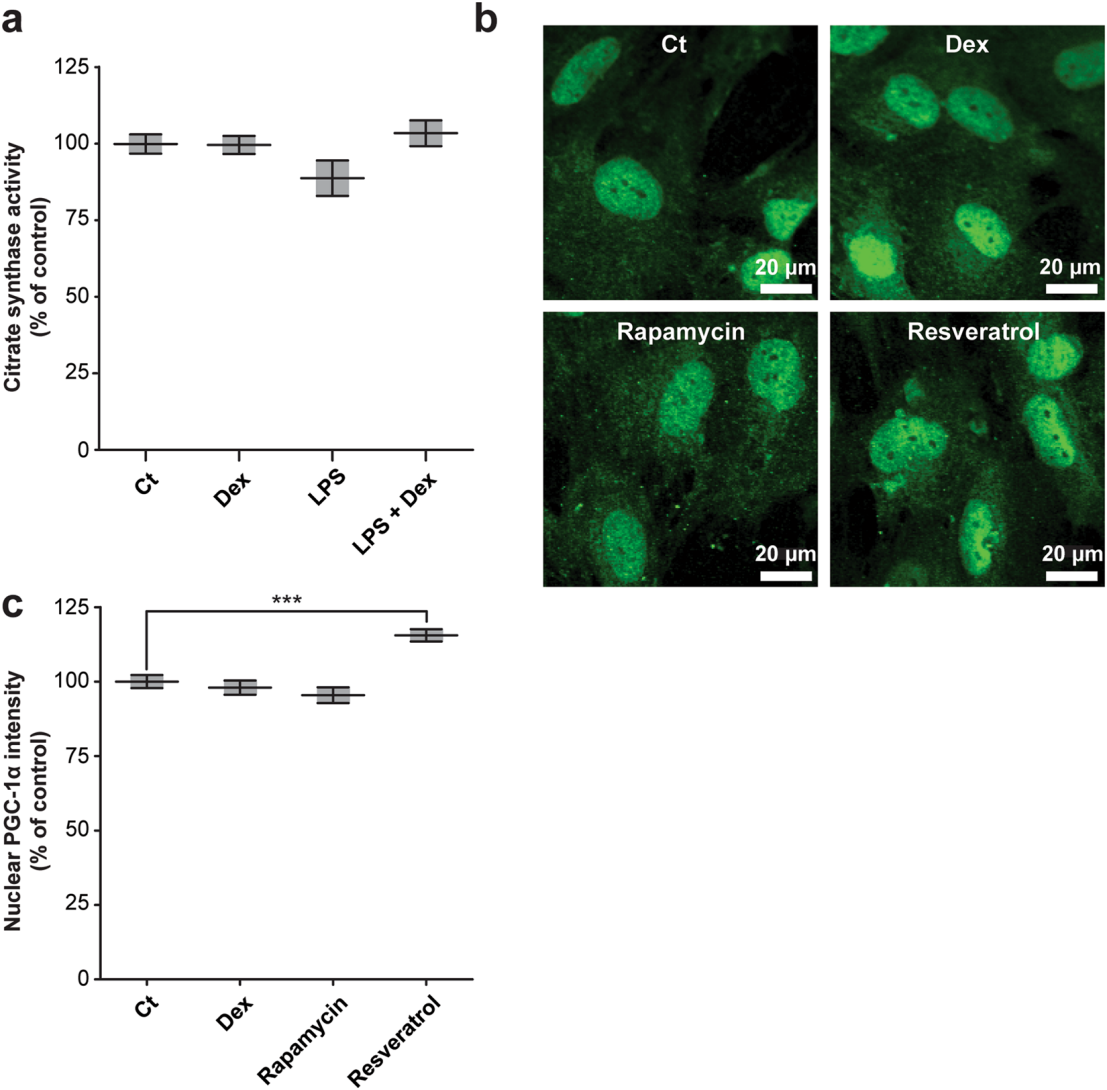
Mitochondrial mass and PGC-1α expression are not affected by dexamethasone. (**a)** Following LPS and/or dexamethasone treatment (Fig. 1a), cells were harvested and citrate synthase activity was measured spectrophotometrically as a measure of mitochondrial mass. Values were corrected for cellular protein and normalised to control (Ct): 362 ± 28 U·mg protein^−1^. Statistic analysis: one-way ANOVA with Tukey’s post-hoc analysis: no statistical differences were observed. Mean ± SEM, n = 3 independent experiments. **(b)** The expression of peroxisome proliferator-activated receptor gamma (PPARγ) coactivator - 1α (PGC-1α), a master regulator of mitochondrial biogenesis, was measured using immunocytochemistry after 4 hours dexamethasone treatment (10 µM, Dex). In addition, cells were incubated for 48 hours with rapamycin (500 nM) or resveratrol (10 µM) as known negative and positive regulators of PGC-1α and mitochondrial mass **(c)** The nuclear PGC-1α expression was quantified using a nuclear mask based on 4′,6-diamidino-2-phenylindole (DAPI) staining. Values were normalised to control (Ct): 15,900 ± 370 arbitrary intensity units. Statistic analysis: one-way ANOVA with Dunnett’s post-hoc analysis: ***p < 0.001. Mean ± SEM, n ≥ 253 individual cells analysed in n = 3 independent experiments.

**Figure 6:**
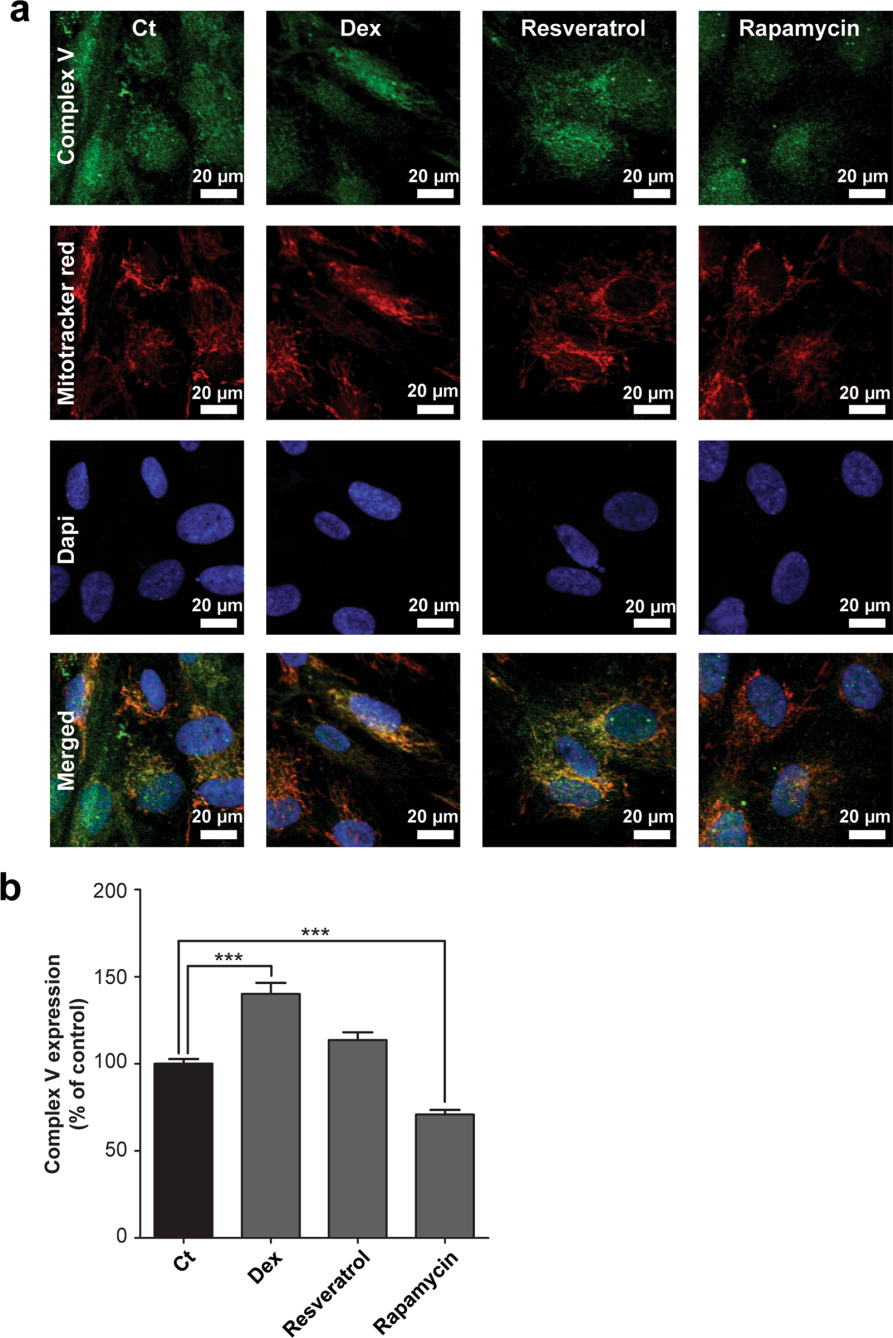
Dexamethasone increases OXPHOS complex V expression. (**a)** The expression of OXPHOS complex V (CV) was measured using immunocytochemistry after 4 hours dexamethasone treatment (10 µM, Dex). In addition, cells were incubated for 48 hours with rapamycin (500 nM) or resveratrol (10 µM) as known negative and positive regulators of mitochondrial mass. Mitochondria were stained with mitotracker red and nuclei with DAPI to determine the cellular localisation. **(b)** The mitochondrial CV expression was quantified using a mitochondrial mask based on the mitotracker red staining. Values were normalised to control (Ct): 40.4 ± 1.1 arbitrary intensity units/mitochondrial pixel. Statistic analysis: one-way ANOVA with Dunnett’s post-hoc analysis: ***p < 0.001. Mean ± SEM, n ≥ 89 individual cells analysed in n = 3 independent experiments.

**Figure 7:**
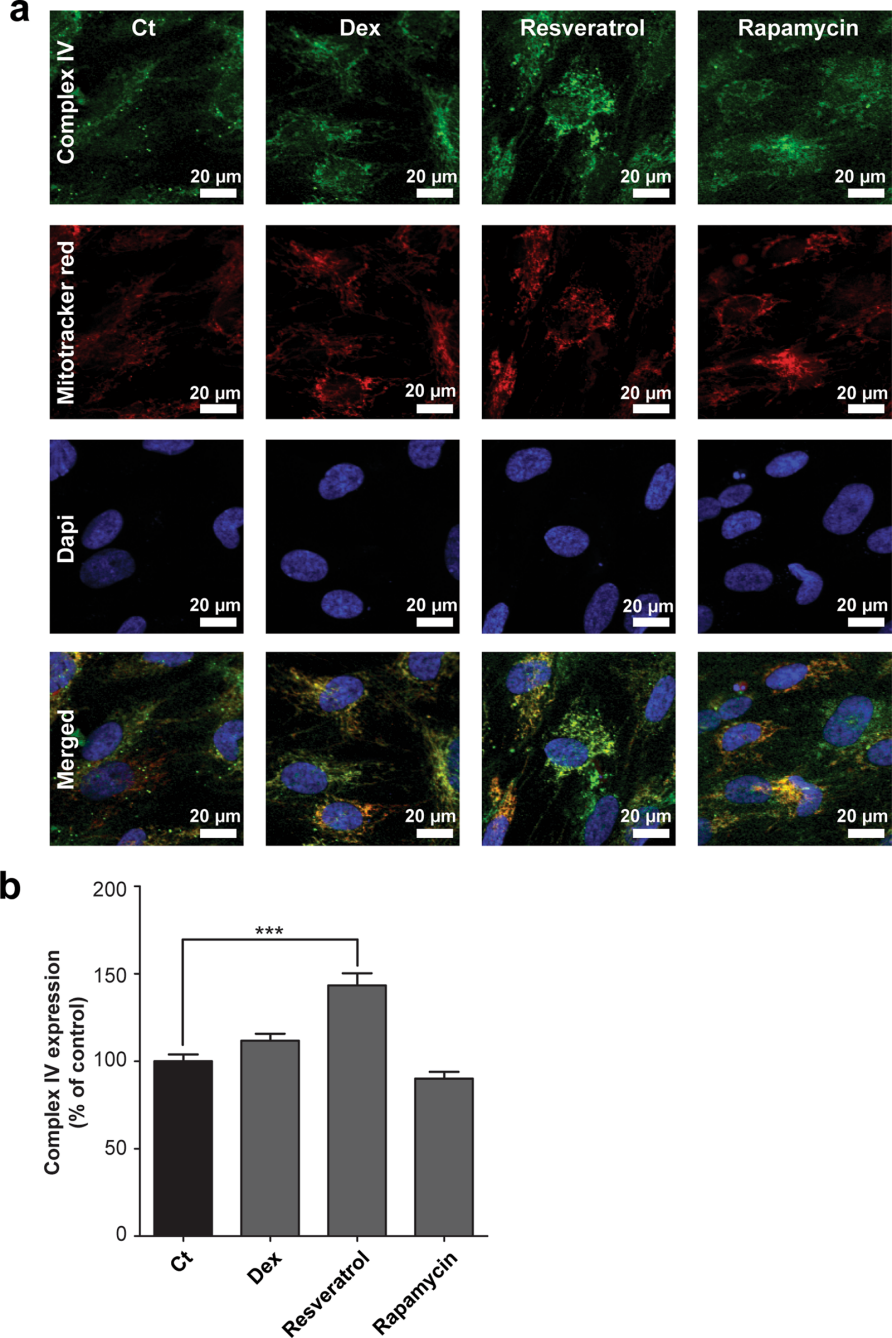
Dexamethasone does not affect the OXPHOS complex IV expression. (**a)** The expression of OXPHOS complex IV (CIV) was measured using immunocytochemistry after 4 hours dexamethasone treatment (10 µM, Dex). In addition, cells were incubated for 48 hours with rapamycin (500 nM) or resveratrol (10 µM) as known negative and positive regulators of mitochondrial mass. Mitochondria were stained with mitotracker red and nuclei with DAPI to determine the cellular localisation. **(b)** The mitochondrial CIV expression was quantified using a mitochondrial mask based on the mitotracker red staining. Values were normalised to control (Ct): 33.2 ± 1.5 arbitrary intensity units/mitochondrial pixel. Statistic analysis: one-way ANOVA with Dunnett’s post-hoc analysis: ***p < 0.001. Mean ± SEM, n ≥ 89 individual cells analysed in n = 3 independent experiments.

Respiratory inhibition combined with a decreased mitochondrial membrane potential after LPS exposure could potentially be explained by a direct inhibition of one of the OXPHOS complexes. We could, however, not detect inhibition of LPS on the enzyme activity of the individual CI to CV activities (Fig. 4). Surprisingly, CV enzyme activity was enhanced upon dexamethasone treatment (123 ± 10%, p = 0.046), and, but not significantly, when cells were co-exposed to LPS and dexamethasone (Fig. 4). The increased CV activity could provide an explanation for the increased mitochondrial membrane potential, when the complex runs into reverse mode (*i.e.* pumps electrons from the mitochondrial matrix to the intermembrane space at the expense of ATP). Besides an increase in the intrinsic enzyme activity, induction could also be due to an enhanced CV expression. However, citrate synthase activity, a marker of mitochondrial mass, did not change upon dexamethasone or LPS exposure (Fig. 5a). To exclude a role of mitochondrial biogenesis, the expression of the master regulator of this process, PGC-1α, was investigated (Fig. 5b)^26^. Resveratrol, a positive control, induced nuclear PGC-1α expression, but no significant changes were found after four hours dexamethasone treatment (Fig. 5c). Using immunocytochemistry, we could confirm increased CV expression per mitochondrial pixel (Fig. 6; 140 ± 6%, p = 0.0001) that was reduced by rapamycin, a mammalian target of rapamycin (mTOR)-dependent mitophagy inducer. To investigate whether the increased expression was specific for CV, CIV expression was determined (Fig. 7), which was up-regulated by resveratrol but not by dexamethasone (Fig. 7c).

### Dexamethasone reverses LPS-induced cellular alkalisation

**Figure 8:**
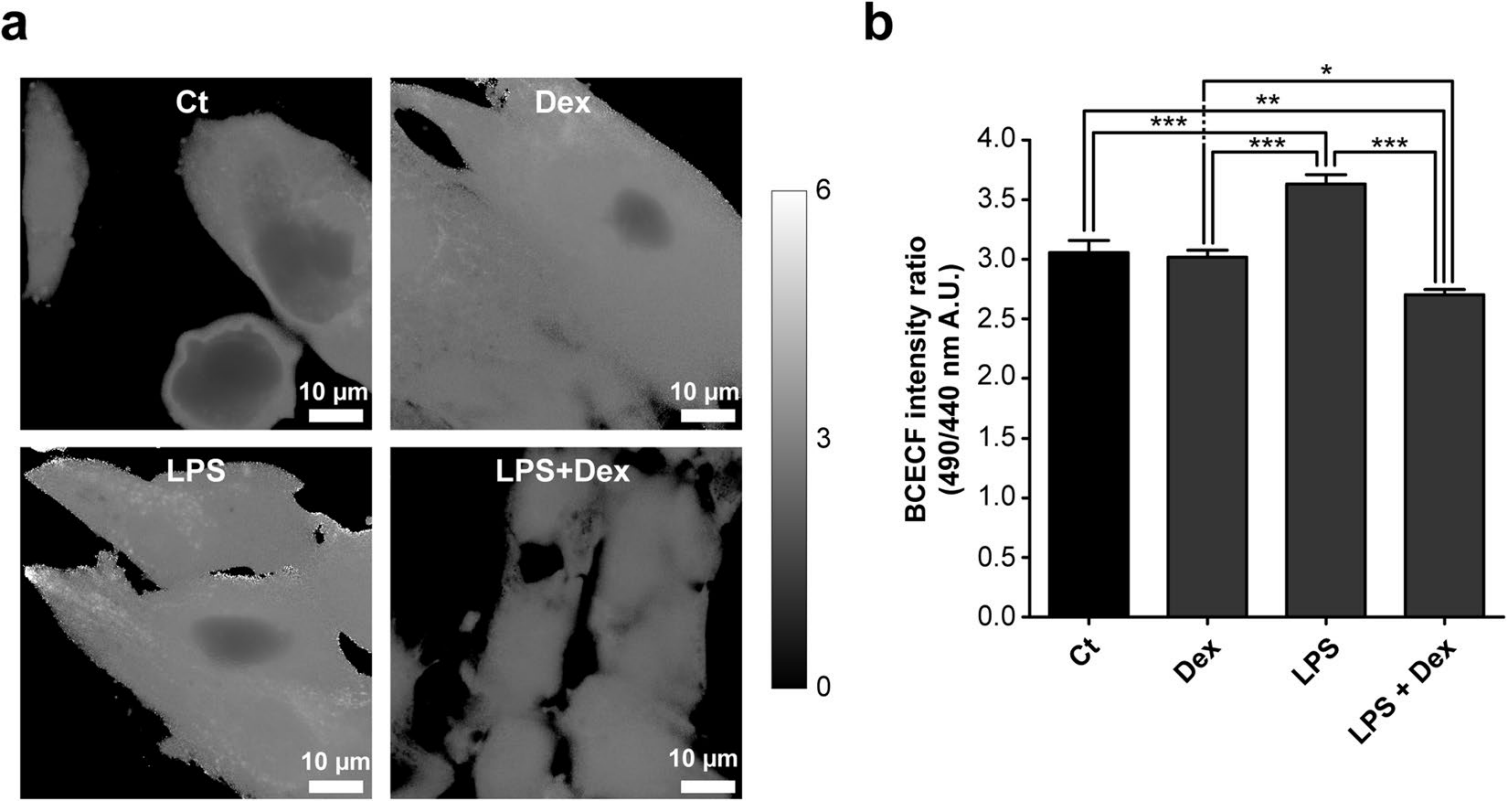
LPS-induced endotoxaemia alkalisation of the intracellular pH is reversed by dexamethasone co-treatment. (**a)** Intracellular pH was measured after LPS and/or dexamethasone exposure (Dex) (Fig. 1a) using the ratiometric probe 2′,7′-bis-(2-carboxyethyl)-5-(and-6)-carboxyfluorescein acetoxy methyl ester (BCECF). pH levels were determined using ratio images, which were obtained by dividing the 490 nm by the 440 nm image, **(b)** and quantified on a single cell level. Statistic analysis: one-way ANOVA with Tukey’s post-hoc analysis to compare to vehicle control (Ct): *p < 0.05, **p < 0.01, ***p < 0.001. Mean ± SEM, n ≥ 77 individual cells analysed in n = 3 independent experiments.

The increased CV expression could potentially be explained as a compensatory mechanism of the reduced intracellular pH upon dexamethasone treatment, as an acidified milieu is known to inhibit activity of the complex^27, 28^. Indeed, the cellular pH measured as 2′,7′-bis-(2-carboxyethyl)-5-(and-6)-carboxyfluorescein acetoxy methyl ester (BCECF) ratio increased after LPS exposure, indicative of alkalisation (Fig. 8a,b; 114 ± 2%, p = 0.00000089). This pH increase is in line with the decreased mitochondrial membrane potential (Fig. 3e), which depends on the ΔpH between the matrix (~7.8) and intermembrane space (~7.0). The mitochondrial outer membrane is permeable for protons, thus the pH of the intermembrane space will be equal to the cytosolic pH. A raise in intracellular pH will, therefore, cause dissipation of the ΔpH and consequently the mitochondrial membrane potential. When co-exposed to dexamethasone, a small but significant cellular acidification was found (Fig. 8b; 87 ± 2%, p = 0.0055). As mentioned, this will result in a minor inhibition of CV, which contributes to the restoration of the LPS-induced decrease in mitochondrial function.

## Discussion

AKI associates with high mortality rates of approximately one out of three intensive care patients^29^, as efficient treatment modalities are absent. Due to the pivotal role of mitochondria in AKI outcome, restoration of their function is expected to have great therapeutic potential^3^. We confirmed their involvement in experimental inflammatory conditions using human ciPTEC. We also demonstrated that dexamethasone attenuates the LPS-induced inflammatory response, illustrated by a reduced cytokine excretion. Simultaneously, dexamethasone induced an intracellular acidification, which coincided with its beneficial effects on LPS-induced mitochondrial dysfunction, including decreased mitochondrial respiration and membrane potential and increased ROS generation. Previously, AKI-associated mitochondrial mechanisms were predominantly attributed to a decreased metabolic capacity, such as an increased ROS generation, decreased mitochondrial biogenesis and mitochondrial permeability pore opening, eventually leading to apoptosis^3, 20^.

Although dexamethasone-induced changes in intracellular pH have not been associated with the beneficial effects in AKI, intracellular acidification has been observed in PTEC. This is explained by a stimulating effect on the basolateral sodium-bicarbonate (Na^+^:HCO_3_^−^) co-transporter (NBC1) and the sodium-proton (Na^+^, H^+^) exchanger (NHE3) at the luminal side^30, 31^, which together with Na^+^, K^+^-ATPase activity are responsible for the renal bicarbonate reabsorption under physiological conditions^32^. Although the acid-base balance determining the intracellular pH is regulated by many mechanisms either providing buffering power of acid or base transport, one possible mechanism by which dexamethasone could induce the observed slight intracellular acidification is by the previously illustrated increase in the transporter activity of NBC1 and NHE3 and increased NBC1 expression^30^. Combined with a stoichiometry of 1:3 for the Na^+^:HCO_3_^−^ efflux, the net effect of dexamethasone causes a drop in the intracellular HCO_3_^−^ concentration and consequently in the pH^30, 31, 33^. However, NHE3 expression has been demonstrated to increase upon LPS exposure in monocytes^34^ and leads to an extracellular acidification in proximal tubule cells^35^. This is associated with inflammatory conditions and decreased cytokine production rates in epithelial cells^36^, illustrating the complex interplay of the different pathways affected by LPS and dexamethasone, and the need to further investigate the exact molecular mechanisms relevant to the observed pH effects.

The drop in intracellular pH after co-treatment with dexamethasone does provide an explanation for the observed up-regulation of mitochondrial CV expression, as the slightly acidified environment is known to inhibit CV^27, 28^, and can also be observed in iron- and ethanol-induced mitochondrial dysfunction. Consequently, this can be considered as a more general response to mitochondrial damage^37, 38^. In addition, our results provide an explanation for the previously observed increased expression of CV and the adenine nucleotide translocator^39^, as both are inhibited by a low intracellular pH^27, 28^. The latter also emphasises the important role of the cellular pH in the observed mitochondrial responses after LPS and dexamethasone treatment.

In addition, dexamethasone is expected to have other beneficial effects on the progression of AKI. Dexamethasone has been demonstrated to increase the renal microvascular oxygenation, which will improve the respiratory capacity while increasing the oxygen availability^18^. Although equivocal results were obtained, dexamethasone is also expected to decrease the iNOS-dependent NO formation, which will lead to attenuate its vasodilatory action and formation of nitrogen radicals^17, 18^. All studies did, however, demonstrate a decreased iNOS expression, which may indicate an additional source of oxidative stress. Mitochondria could account for this additional source as they are a major ROS generation site containing 11 ROS-producing enzymes (mainly by complex I, II, and III, pyruvate dehydrogenase and 2-oxoglutarate dehydrogenase)^24^. Although our results indicate a central role of mitochondria in the beneficial effects of dexamethasone, which is supportive for its ROS-lowering activity, we cannot exclude other dexamethasone effects. For example, dexamethasone appeared to be an inhibitor of NADPH oxidase (NOX)-2-dependent ROS generation as well^40^.

Until now almost all *in vivo* and patient studies showed beneficial effects of dexamethasone in a prophylactic setting^12, 15, 17, 20^. This warrants further investigation of the potential beneficial effects of dexamethasone treatment at the manifestation of AKI in animal models and patients. However, its narrow therapeutic window, in general, limits dexamethasone intervention. This is probably also the main reason for the equivocal results obtained in clinical trials, whereas our findings with dexamethasone on cellular morphology and ROS levels may indicate beneficial effects of low-dose treatments opposed to no or detrimental effects of high-dose regimens^12, 13, 17, 18^. Future research should provide better insight into the balance between efficacy and safety of dexamethasone therapy. Alternatively, novel pharmaceutical interventions could be investigated, which also target the cellular pH without impacting the adverse mechanisms activated upon high-dose dexamethasone treatment.

In conclusion, we provide mechanistic insights into the observed beneficial effects of dexamethasone in AKI and provide evidence for a key role of mitochondria in the disease. Furthermore, we demonstrated the importance of this mechanism for the development of inflammatory conditions, in addition to mitochondria-mediated apoptosis in AKI^3, 39^. Modulating the pH of proximal tubule might be a novel therapeutic approach for treatment of patients suffering from septic AKI.

## Materials and Methods

### Chemicals and cell culture materials

Chemicals were purchased from Sigma-Aldrich unless stated otherwise. Cell culture plates were purchased from Greiner Bio-One.

### Cell culture and experimental design

Human conditionally immortalised PTEC isolated from kidney tissue (ciPTEC-T1^23^) were cultured in phenol-red free DMEM-HAM’s F12 medium (Lonza) containing 10% (*v/v*) FCS (Greiner Bio-One), insulin (5 µg/ml), transferrin (5 µg/ml), selenium (5 µg/ml), hydrocortisone (36 ng/ml), EGF (10 ng/ml) and tri-iodothyronine (40 pg/ml) (complete PTEC medium^23^). In all experiments, cells were seeded using a density of 25,000 cells/cm^2^ on uncoated surfaces, unless stated otherwise. Cells were maintained at 33 °C, 5% (*v/v*) CO_2_ for 24 hours, to proliferate and subsequently transferred to 37 °C, 5% (*v/v*) CO_2_ for 7 days to mature. On day 6, cells were exposed to control medium (complete PTEC medium) or medium supplemented with LPS (10 µg/ml, ref. 41) for 24 hours at 37 °C, 5% (*v/v*) CO_2_ to induce endotoxaemia (see also Fig. 2a). After 20-hour incubation, control or LPS-treated cells were co-incubated in the presence or absence of dexamethasone (10 µM).

### Enzyme-linked Immuno Sorbent Assay

The cytokine production of interleukin-6 (IL-6; #DY206, R&D systems) and -8 (IL-8; #DY208, R&D systems) in ciPTEC under control or inflammatory conditions in the presence or absence of dexamethasone was quantified using Enzyme-Linked Immuno Sorbent Assays (ELISAs) as previously described by Schophuizen *et al*.^42^. The optical density of each well was measured using an iMark Microplate reader (BioRad) set to 460 nm and were background corrected using readings obtained at 540 nm.

### High-resolution respirometry

Cellular and mitochondrial respiration was measured at 37 °C using a two-chamber Oxygraph-2k equipped with Datlab 5 recording and analysis software (Oroboros Instruments), as described previously^43–45^. To determine the P:O ratio we slightly modified the previously described approach^45, 46^. For cellular respiration measurements, 1.5·10^6^ cells were resuspended in mitochondrial respiration medium MiR05 (Oroboros Instruments) and transferred to the chambers of an Oxygraph-2k. After digitonin permeabilisation (10 µg/1·10^6^ cells) of the cell membrane, malate (2 mM) and glutamate (10 mM) were added to determine the STATE 4 respiration. STATE 3 respiration was obtained by the addition of ADP (220 nM), after which the oxygen consumption rate could decrease to the level of STATE 4 respiration, indicating a complete conversion of ADP in ATP. The P:O ratio was calculated dividing the amount of ADP used by the amount of oxygen used by the mitochondria to use all the ADP. Next, the mitochondrial complex-specific respiration was determined using complex-specific substrates and ADP (4 mM). Glutamate (10 mM) and malate (2 mM) were used as CI substrates, succinate (10 mM) for CII and ascorbate (2 mM) plus TMPD (0.5 mM) as CIV substrates. The glycerol-3-phosphate dyhydrogenase (G3PDH)-driven respiration was measured in the presence of glycerol-3-phosphate (20 mM) and flavine adenine dinucleotide (10 µM) and was terminated by antimycin A (2.5 µM). To inhibit CI and CII, rotenone (0.5 µM) and atpenin A5 (50 nM, Enzo Life Sciences) were added, respectively. Finally, the integrity of the mitochondrial outer membrane was tested using 10 µM cytochrome *c* (respiratory rate increase should be less than 10%)^47^.

### Mitochondrial membrane potential analysis

Cells were seeded in 35 mm Fluorodishes (World Precision Instruments GmbH) and after exposure loaded with loaded with tetramethylrhodamine methyl ester (100nM) (TMRM, Thermo Fisher Scientific) for 25 minutes at 37 °C, 5% (*v/v*) CO_2_. Next, cells were washed twice using Krebs-Henseleit buffer supplemented with HEPES (10 mM, KHH) (pH 7.4), and images were captured using a temperature-controlled chamber connected to an inverted microscope (Axiovert 200M, Carl Zeiss) using a x63, 1.25 NA Plan NeoFluor oil immersion objective. As a positive control, the known uncoupling agent carbonyl cyanide-4-(trifluoromethoxy)phenylhydrazone (FCCP) was used at the end of each measurement. Images were corrected for background and uneven illumination followed by analysis where images were masked with a binarised image for mitochondrial morphology using Image Pro Plus software (version 6.3, Media Cybernetics) as previously described^48^.

### Reactive oxygen species generation analysis

To measure cellular ROS, ciPTEC-T1 were seeded in flat bottom 96-well black/clear plates (Corning). ciPTEC-T1 cells were loaded with 5-(and-6)-chloromethyl-2′,7′-dichlorodihydrofluorescein diacetate, acetyl ester (CM-H_2_DCFDA, 10 µM, Thermo Fisher Scientific) in KHH buffer for 20 minutes at 37 °C, 5% (*v/v*) CO_2_. Mitochondrial ROS production was detected using hydroethidium (10 µM, Thermo Fisher Scientific). Subsequently, cells were washed twice using KHH buffer and imaging was performed using a BD Pathway 855 high-throughput microscope (Becton Dickinson) for endpoint CM-DCF intensity measurements. Images were background corrected and average CM-DCF or hydroethidium intensity per cellular pixel was determined Image Pro Plus software (version 6.3, Media Cybernetics). CM-DCF oxidation rates were determined using a Perkin Elmer Victor X3 fluorescent plate reader. CM-DCF intensity was read for 17 minutes and subsequently corrected for the basal intensity of each well. Next, the oxidation rate was determined using the slope of the linear part of the plotted CM-DCF intensity.

### OXPHOS complex and citrate synthase activities

Approximately 6·10^6^ exposed ciPTEC-T1 cells were harvested and resuspended in Tris-HCl buffer (10 mM). Next, samples were pottered in the presence of sucrose (215 mM) to obtain a homogenous fraction. Samples were centrifuged for 10 minutes at 600 *g* to remove cellular debris. The supernatant containing mitochondria was centrifuged for 10 minutes at 14,000 *g*. The pellet was resuspended in Tris-HCl buffer (pH 7.6), snap frozen in liquid N_2_ and stored at -80 °C until usage. The catalytic capacity of the OXPHOS complexes was measured using a spectrophotometric method, as previously described^49^. Citrate synthase activity was determined simultaneously as described previously^50^. The catalytic activity of CI - IV and citrate synthase were corrected for mg cellular protein.

### Cytosolic pH measurements

ciPTEC-T1 were seeded in 35 mm Fluorodishes (World Precision Instruments GmbH) and cultured accordingly. Cells were loaded with the cytosolic pH-sensitive reporter molecule BCECF-AM (5 µM, 2′,7′-bis-(2-carboxyethyl)-5-(and-6)-carboxyfluorescein acetoxy methyl ester) (Thermo Fisher Scientific) in KHH buffer and incubated for 15 minutes at 37 °C. Cells were washed three times using KHH buffer and imaging was performed using an inverted microscope (Axiovert 200M, Carl Zeiss). BCECF fluorescence was sequentially excited at the isosbestic point (440 nm) (100 ms) and at the H^+^-sensitive wavelength (490 nm) (100 ms). Fluorescent images were captured at 530 nm emission wavelength. Images were analysed using Image Pro Plus software (version 6.3, Media Cybernetics). After background correction, the 490/440 emission ratio was used to quantify cellular pH^45, 51^.

### Immunocytochemistry

We used µ-slide 8 well chambers (Ibidi GmbH) coated with human collagen IV (50 µg/ml in Hank’s Balanced Salt Solution (HBSS), Thermo Fisher Scientific) to allow the cells to attach. As controls, cells were exposed to resveratrol (10 µM) for 48 hours at 37 °C, 5% (*v/v*) CO_2_ to induce the expression of PCG-1α, COX (CIV) and CV, whereas rapamycin (500 nM) was used for 48 hours to attenuate the expression of the proteins of interest as it is a known mTOR-dependent mitophagy inducer. First, exposed ciPTEC were incubated using mitotracker red (400nM, Thermo Fisher Scientific) for 30 minutes at 37 °C, 5% (*v/v*) CO_2_. Next, cells were fixed using 2% (*w/v*) paraformaldehyde in HBSS supplemented with 2% (*w/v*) sucrose for 5 minutes and permeabilised using 0.3% (*v/v*) Triton X-100 in HBSS for 10 minutes, all at room temperature (rT). To prevent a-specific binding of antibodies, cells were blocked using 2% FCS (*v/v*), 2% (*w/v*) BSA fraction V (Roche) and 0.1% (*v/v*) Tween-20 in HBSS for 30 minutes. Next, cells were incubated against PCG-1α (1:50 dilution in block solution; clone 3G6; Cell Signaling Technology), COX (1:100 dilution in block solution; ab14705, Abcam) and CV (1:100 dilution in block solution; clone 7H10BD4F9, Mitosciences, Abcam) for 1 hour at rT. Subsequently, cells were incubated with goat-anti-rabbit- or goat-anti-mouse-Alexa488 conjugate (1:200, Abcam) and finally nuclei were stained using DAPI (300 nM, Thermo Fisher Scientific) for 5 minutes at rT. Images were captured using the Olympus FV1000 Confocal Laser Scanning Microscope (Olympus) and the Olympus software FV10-ASW version 1.7. Images were analysed using Image Pro Plus software (version 6.3, Media Cybernetics). For both COX and CV images, background was subtracted and subsequently a mitochondrial binary mask was created using the mitotracker red images. Next, the mitochondrial mask was combined with the COX or CV images and the intensity per mitochondrial pixel was quantified. The PGC-1α images were combined with a nuclear binary mask instead of a mitochondrial mask, which was made using the DAPI images.

### Data analysis

All data are expressed as mean ± SEM of multiple independent replicates as indicated. Statistical analysis was performed using GraphPad Prism version 5.03. A one-way or two-way ANOVA analysis was performed followed by the appropriate post-hoc analysis as indicated.

### Data availability

The datasets generated and/or analysed are available on reasonable request. All experiments were performed in accordance with relevant guidelines and regulations.

## Electronic supplementary material

RusselSupplementaryInformation
