# Supplementary material for: Mild intracellular acidification by dexamethasone attenuates mitochondrial dysfunction in a human inflammatory proximal tubule epithelial cell model
Source: Sci Rep. 2017 Sep 6;7:10623. doi: 10.1038/s41598-017-10483-y (PMC5587643; doi:10.1038/s41598-017-10483-y)
Supplement: Supplementary file 1 — RusselSupplementaryInformation [file 41598_2017_10483_MOESM1_ESM.pdf]

SUPPLEMENTARY INFORMATION

Mild intracellular acidification by dexamethasone attenuates mitochondrial dysfunction in a human inflammatory proximal tubule epithelial cell model

Schirris T.J.J., Jansen J., Mihajlovic M., van den Heuvel L.P., Masereeuw R., Russel F.G.M.

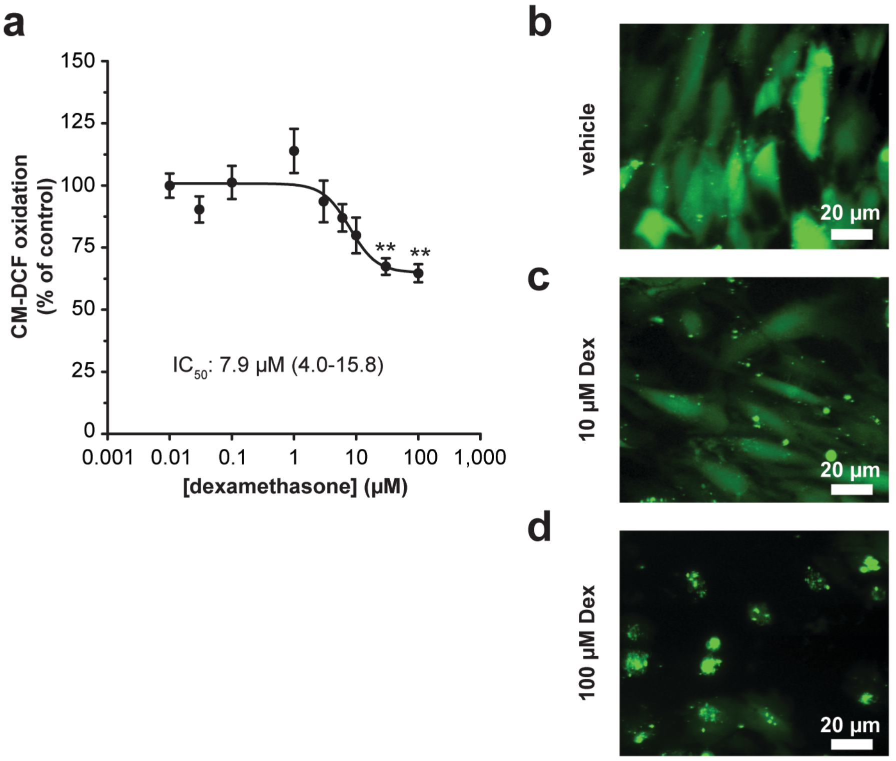

Supplementary figure 1 I Low dexamethasone concentrations decreases reactive oxygen species and high concentrations are cytotoxic. (a-d) ciPTEC cells were treated for 24 hours with various dexamethasone (Dex) concentrations after which the generation of reactive oxygen species was determined using 5-(and-6)-chloromethyl-2′,7′-dichlorodihydrofluorescein diacetate, acetyl ester (CM-DCF). **(b-d)** Effects of treatment with **(b)** vehicle, **(c)** 10 μM, or **(d)** 100 μM dexamethasone on the CM-DCF staining pattern and cell morphology were evaluated microscopically. Statistical analysis: one-way ANOVA with Dunnett's post-hoc analysis: **p<0.01. Mean ± SEM, n = 2 independent experiments.
